# Psychological Effects and Associated Factors of COVID-19 in a Mexican Sample

**DOI:** 10.1017/dmp.2020.215

**Published:** 2020-06-24

**Authors:** Nadia Yanet Cortés-Álvarez, Regino Piñeiro-Lamas, César Rubén Vuelvas-Olmos

**Affiliations:** School of Medicine, University of Colima, Colima, Mexico; School of Medicine, José Martí University, Colima, México

**Keywords:** anxiety, depression, COVID-19, epidemic, psychological distress, stress

## Abstract

**Objectives:**

The coronavirus disease 2019 (COVID 19) is a new viral zoonosis of global concern that could cause psychological sequelae. We examined the levels of psychological distress, anxiety, depression, and stress during the COVID-19 outbreak in a Mexican sample.

**Methods:**

An online survey was applied that collected information on demographic and financial status data, physical status, contact history, knowledge, concerns, and precautionary measures concerning COVID-19. Impact of Event Scale-Revised and Depression, Anxiety, and Stress Scale were included.

**Results:**

A total of 50.3% of respondents rated psychological distress as moderate-severe; 15.7% reported moderate-severe depressive symptoms; 22.6% reported moderate-severe anxiety symptoms; and 19.8% reported moderate-severe stress levels. Female gender, older age, divorced status, lack of confidence related to security of the test, lower satisfaction of health information concerning COVID-19, history of direct or indirect contact with a COVID-19 confirmed case, live with just 1 other person, and spent >9 h/d at home were associated with greater psychological distress and/or higher levels of stress, anxiety, and depression. By contrast, precautionary measures, such as hand hygiene and wearing masks, were associated with lower levels of psychological distress, depression, anxiety, and stress.

**Conclusions:**

COVID-19 outbreak results in considerable psychological effects among the Mexican sample.

Coronavirus disease 2019 (COVID-19) originated in the city of Wuhan, China, on December 31, 2019. Since its discovery, a rapid community, regional, and international spread has occurred with exponential growth in cases and deaths.^1^ The COVID-19 outbreak was confirmed to have reached Mexico on February 28, and on March 30, the Mexico government declared a national health emergency.^2^ In the face of a health crisis, not seen in several years, response efforts by the government in coordination with the Secretaría de Salud (Ministry of Health) of Mexico have been swift, implementing a series of preventions, actions, and control infections in the country, including extension of school vacation period, home office, restriction of massive events, suspension of “nonessential activities” in all economic sectors, and the recommendation of home confinement for the general population.^3^ Some negative implications that COVID-19 has had in Mexico include the generation of panic buying and shoplifting, which in turn has led to the eventual shortage of antibacterial hand gel and face masks,^4^ a fall in the price of fuel,^5^ and the temporary or definitive closure of companies.^6^ In fact, in the past month, 346,878 formal jobs were lost in Mexico between March 13 and April 6.^7^

COVID-19 not only threatens people’s physical health, but also could affect people’s mental health, as many theories indicate. For example, according to Behavioral Immune System theory,^8^ people show emotions, such as aversion and anxiety, for self-protection.^9,10^ Moreover, stress theory^11^ and perceived risk theory^12^ mention that public health emergencies trigger negative emotions, which may reduce the immune function of people and destroy the balance of their normal physiological mechanisms.^13^ In support, previous studies have shown the immediate psychological sequelae of a pandemic, as occurred with severe acute respiratory syndrome (SARS) and H1N1, include stress, poor sleep, depressed mood, weepiness, nightmares, and poor concentration.^14^ Furthermore, it has also been shown that people experience fear of falling sick or dying themselves, feelings of helplessness, irrational nervousness, loneliness, and stigma.^15,16^

Also, it has been identified that certain groups are more vulnerable to high psychological distress during a pandemic. Taylor et al. reported that, during an outbreak of equine influenza, younger people, those with no formal educational qualifications, and those with financial dependence on an industry facing a crisis are significantly predisposed to high psychological distress. Finally, the authors also identified that those with 1 child had a 1.2 times higher risk of high psychological distress than those with no children.^17^ For their part, during pandemic influenza, Jacobs et al. found that individuals with poor self-rated health reported greater risk perception and concern for self and others than individuals with good self-rated health.^18^ Finally, di Giuseppe et al. reported that people with lower socioeconomic status and lower education showed significantly higher risk perception during avian influenza.^19^

Therefore, it is essential to understand the potential psychological effects caused by COVID-19 in a timely manner. Based on our understanding, there is no information on the psychological distress and mental health of Mexican populations concerning COVID-19. This is especially pertinent with the uncertainty surrounding an outbreak of such unparalleled magnitude in our country. Therefore, this study aims to examine the levels of psychological distress (anxiety, depression, and stress) and identify risk factors contributing to worse outcome during the COVID-19 outbreak in a Mexican sample. We hypothesized that most of the respondents will show severe psychological distress and moderate depressive, anxiety, and stress levels caused by COVID-19. In addition, age, gender, and financial status will be factors contributing to worse outcomes.

## METHODS

### Participants and Procedure

We carried out a cross-sectional study design. As the Mexican Government recommended home confinement for the general population, an anonymous online questionnaire in Spanish was applied through an online survey platform (“Google Forms”, Google Inc., CA). The online survey was first disseminated to university students, and they were encouraged to pass it on to others. A snowball sampling strategy, focused on recruiting the general public living in the whole country during the epidemic of COVID-19, was used. This study was conducted in compliance with the Norma Oficial Mexicana-012-SSA3-2012, Declaration of Helsinki and the Ethics Committee of Universidad José Martí (Approval number 2020-3). Informed consent was obtained from all participants included in the study. Data collection was carried out from March 30 to April 5, that is, immediately after a national health emergency was declared in Mexico.

### Survey

We reviewed a previous survey on the psychological distress of COVID-19 performed in China.^20^ In addition, we included questions related to employment status during the COVID-19 outbreak. The questionnaire consisted of 70 items (about the past 14 days) that covered 8 areas: demographic data, financial status, physical health, contact history, knowledge and concerns, precautionary measures, psychological distress, and mental health status (more details see Appendix 1, online only).

The psychological distress was examined using the Impact of Event Scale-Revised (IES-R). The IES-R is a self-administered questionnaire that has been applied to the Mexican sample for determining the extent of psychological distress after traumatic and/or stressing experiences.^21^ In addition, it has been used in a recent study for determining the extent of psychological distress during COVID-19.^20^ The questionnaire was composed of 22 multiple choice items. The responses are rated on a 5-point scale ranging from 0 (“not at all”) to 4 (“completely agree”). The total IES-R score was used to perform the inferential statistics, whereas for descriptive statistics, the total IES-R score was divided into normal (0–23), mild (24–32), moderate (33–36), and severe psychological distress (>37).^22^

The mental health status was assessed by the Depression, Anxiety, and Stress Scale (DASS-21). This scale has been used previously in research related to outbreaks as H1N1,^23^ SARS,^24^ and recently, in COVID-19 outbreak in china (20). In addition, DASS-21 scale is considered as a reliable and valid measure in assessing mental health in the Mexican population.^25^ This 21-item questionnaire aims to measure depression, anxiety, and stress subscales. Response options are on a 4-point scale (0 = did not apply to me at all and 3 = applied to me most of the time). Questions 3, 5, 10, 13, 16, 17, and 21 formed the depression subscale. The total depression subscale score was divided into normal (0–9), mild depression (10–12), moderate depression (13–20), severe depression (21–27), and extremely severe depression (28–42). Questions 2, 4, 7, 9, 15, 19, and 20 formed the anxiety subscale. The total anxiety subscale score was divided into normal (0–6), mild anxiety (7–9), moderate anxiety (10–14), severe anxiety (15–19), and extremely severe anxiety (20–42). Questions 1, 6, 8, 11, 12, 14, and 18 formed the stress subscale. The total stress subscale score was divided into normal (0–10), mild stress (11–18), moderate stress (19–26), severe stress (27–34), and extremely severe stress (35–42).^20^

### Statistical Analysis

Descriptive statistics was used for categorical variables, such as sociodemographic characteristics, financial status, physical status, contact history, knowledge and concerns, and precautionary measures. Univariate generalized linear with a main effects model (linear regression) was used to measure the associations between sociodemographic characteristics, financial status, physical status, contact history, knowledge and concerns, and precautionary measures with regard the total IES-R score and DASS-21 subscales scores. Associations are presented using beta coefficients, confidence intervals, and *P*-values. The significance level was set at 0.05. Analyses were performed in IBM SPSS Statistics (version 25.0).

## RESULTS

A total of 1105 persons from 32 states in Mexico were included in the study (more details see Appendix 2, online only) from a convenience sample of 1109 respondents (response rate = 99.63). Sociodemographic characteristics of the final sample of respondents are presented in Table 1. The majority of respondents were female (62.1%), aged 18 to 28 y (68.7%) (SD = 10.46), single (71.1%), with a household size of >3 people (51.6%), without children (67.4%), and well educated (71.2% ≥ college degree).

**TABLE 1:** Sample Characteristics

Parameter	*N*	Percent
Gender		
Male	418	37.9
Female	686	62.1
Age (years)		
18 to 28	759	68.7
29 to 39	199	18.0
40 to 50	89	8.1
51 a 61	47	4.3
>62	10	0.9
Level of education		
Primary school	4	0.4
Secondary school	41	3.8
High school	263	24.5
College	672	62.6
Specialty	29	2.7
Master	54	5.0
Doctorate	10	0.9
Marital status		
Single	785	71.1
Common-law marriage	65	5.9
Married	210	19.0
Divorced	35	3.2
Widowed	9	0.8
Parental status		
With children	292	26.4
Not children	812	73.6
Household size		
1	85	7.7
2	181	16.4
3	268	24.3
>3	570	51.6

Abbreviations: β, beta coefficient; CI, confidence interval; *N* = sample size.

### Psychological Distress and Mental Health

The psychological distress of COVID-19 outbreak, measured using the IES-R scale, revealed a sample mean score of 26.07 (SD = 19.33). Of the total respondents, 33% reported minimal psychological distress, 16.7% rated mild psychological distress, and 50.3% reported moderate or severe psychological distress. About the DASS 21 scale, who assessed mental health, revealed a sample mean score of 13.38 (SD = 4.51). Results showed that 15.7% reported moderate to severe depressive symptoms, 22.6% of respondents reported moderate to severe anxiety symptoms, and 19.8% reported moderate to severe stress levels.

### Sociodemographic Characteristics, Psychological Distress, and Mental Health

The results of the associations of psychological distress and mental health according to the sociodemographic variables are shown in Table 2.

**TABLE 2:** Association Between Demographic Characteristics, Psychological Impact, and Mental Health Status During the COVID-19 Outbreak

	Impact of Event Scale Revised (IES-R)			Depression, Anxiety, and Stress Scales (DASS-21)								
	IES-R Total		Depression			Anxiety			Stress			
	*β*	95% CI	*P* value	*β*	95% CI	*P* value	*β*	95% CI	*P* value	*β*	95% CI	*P* value
Gender												
Female	7.273	1.1881 to 9.960	0.0001**	1.375	0.3208 to 2.004	0.015*	1.361	-0.3266 to 1.962	0.0001**	1.682	0.3542 to 2.376	0.0001**
Male	Reference			Reference			Reference			Reference		
Age												
18 to 28	12.299	-.035 to 24.6233	0.061	.381	-.034 to .797	0.072	3.607	.404 to 6.810	0.027*	5.236	1.552 to 8.920	0.005*
29 to 39	11.616	-.500 to 23.732	0.70	.259	-.148 to .667	0.213	2.929	-.217 to 6.076	0.068	4.481	.862 to 8.100	0.015*
40 to 50	14.418	2.018 to 26.818	0.019*	.266	-.152 to .684	0.212	3.623	-.100 to 6.345	0.058	3.898	.191 to 7.604	0.039*
51 to 61	7.611	-5.301 to 20.522	0.248	.039	-.395 to .474	0.859	1.432	-1.921 to 4.784	0.403	1.826	-2.030 to 5.683	0.353
>62	Reference			Reference			Reference			Reference		
Level education												
Primary	-6.373	-28.201 to 5.454	0.567	-.324	-1.059 to .410	0.387	-1.271	-6.939 to 4.397	0.660	-4.605	-1.124 to 1.915	0.166
Secondary	8.465	-4.647 to 21.577	0.206	.141	-.301 to .582	0.532	3.061	-.344 to 6.466	0.078	.453	-3.464 to 4.369	0.821
High school	8.764	-3.304 to 20.832	0.155	.092	-.315 to .498	0.659	3.137	.003 to 6.271	0.060	1.449	-2.155 to 5.054	0.431
College	2.892	-9.023 to 14.808	0.634	-.120	-.521 to .281	0.557	1.525	-1.570 to 4.619	0.334	-.301	-3.859 to 3.258	0.869
Specialty	1.343	-12.199 to 14.885	0.846	-.033	-.489 to .423	0.888	2.061	-1.456 to 5.577	0.251	-.428	-4.472 to 3.617	0.836
Master	-2.827	-15.542 to 9.887	0.663	-.117	-.545 to .311	0.592	.340	-2.962 to 3.641	0.840	-1.255	-5.052 to 2.53	0.517
Doctorate	Reference			Reference			Reference			Reference		
Marital status												
Single	1.350	-10.949 to 13.648	0.830	.323	-0.091 to .737	0.127	-.003	-3.197 to 3.191	0.999	.328	-3.345 to 4.002	0.861
Common-law marriage	-2.014	-15.071 to 11.043	0.762	.158	-.281 to .598	0.480	-.313	-3.704 to 3.078	0.856	-.365	-4.265 to 3.535	0.854
Married	-1.295	-13.791 to 11.202	0.839	.232	-.189 to .652	0.281	-.634	-3.879 to 2.611	0.702	-.889	-4.621 to 2.844	0.641
Divorced	2.203	-11.544 to 15.950	0.753	.630	.168 to 1.093	0.008**	.715	-2.855 to 4.284	0.695	1.357	-2.749 to 5.463	0.517
Widowed	Reference			Reference			Reference			Reference		
Parental status												
With children	2.371	-1.219 to 5.962	0.196	.018	-.103 to .139	0.768	-.221	-1.152 to 7.11	0.642	-.089	-1.161 to .983	0.871
Not children	Reference			Reference			Reference			Reference		.
Household size												
1	.079	-.150 to .307	0.500	.122	-.026 to .269	0.107	.797	-.343 to 1.936	0.171	.884	-.427 to 2.195	0.186
2	3.722	.499 to 6.944	0.015*	.076	-.034 to .185	0.174	.852	.008 to 1.696	0.048*	.854	-.117 to 1.824	0.085
3	.076	-.070 to .222	0.309	.016	-.079 to .110	0.744	.007	-.721 to .735	0.984	.334	-.503 to 1.172	0.434
>3	Reference			Reference			Reference			Reference		

Abbreviations: β, beta coefficient; CI, confidence interval.

*P<0.05

** *P*<0.001

#### Gender

Female gender was significantly associated with higher scores on the IES-R scale (B = 7.273; 95% confidence interval [CI] = 1.1881 to 9.960). In the DASS-21 subscales, women were also the ones who showed higher scores in depression (B = 1.375; 95% CI = 0.3208 to 2.004), anxiety (B = 1.361; 95% CI = 0.3266 to 1.962), and stress (B = 1.682; 95% CI = 0.3542 to 2.376).

#### Age

Participants of 40-50 y were significantly associated with higher scores on IES-R scale (B = 14.418; 95% CI = 2.018 to 26.818). About DASS-21 assessment, participants of 18 to 28 y were significantly associated with higher scores in anxiety (B = 3.607; 95% CI = 0.404 to 6.810) and stress subscale (B = 5.236; 95% CI = 1.552 to 8.920). In addition, respondents of 29 to 39 y (B = 4.481; 95% CI = 0.862 to 8.100) and 40 to 50 y (B = 3.898; 95% CI = 0.191 to 7.604) showed a significantly association with higher scores in stress subscale.

#### Marital Status

Divorced status was significantly associated with higher scores in DASS-21 subscale depression (B = 0.630; 95% CI = 0.168 to 1.093). Marital status was not associated with IES-R score.

#### Household Size

Household size of 2 persons was significantly associated with higher scores in DASS-21 subscale anxiety (B = 0.852; 95% CI = 0.008 to 1.696) and higher scores on IES-R scale (B = 3.722; 95% CI = 0.499 to 0.6.944).

### Financial Status, Psychological Distress, and Mental Health

Employed (52.4%) and student (38.8%) status were more frequent for respondents. The most common monthly income was 0-4000 MXN. Most of the employees did not show risk for job loss (56.6%), reduction in working days (58.8%), or reduction in monthly income (52.0%). The financial status was not associated with DASS-21 and IES-R subscale scores (see Table 3).

**TABLE 3:** Association Between Financial Status, Psychological Impact, as Well as Adverse Mental Health Status During the Epidemic

	Impact of Event Scale Revised (IES-R)			Depression, Anxiety and Stress Scales (DASS-21)								
	IES-R Total			Depression			Anxiety			Stress		
	*β*	95% CI	*P* value	*β*	95% CI	*P* value	*β*	95% CI	*P* value	*β*	95% CI	*P* value
Employment status												
Student	2.944	-27.964 to 33.852	0.852	-3.047	-11.280 to 5.187	0.468	-2.069	-9.983 to 5.845	0.608	.314	-9.002 to 9.629	0.947
Employee	-3.345	-19.961 to 13.270	0.693	-2.024	-6.451 to 2.402	0.370	-1.343	-5.597 to 2.912	0.536	-.971	-5.979 to 4.037	0.704
Housewife	-2.964	-19.542 to 13.614	0.726	-2.352	-6.768 to 2.065	0.297	-1.351	-5.596 to 2.893	0.533	-1.121	-6.117 to 3.875	0.660
Unemployed	-5.227	-23.279 to 12.825	0.570	-1.966	-6.775 to 2.843	0.423	-.520	-5.142 to 4.102	0.825	-.724	-6.165 to 4.716	0.794
Retired	-5.540	-23.017 to 11.936	0.534	-1.442	-6.098 to 3.213	0.544	-1.373	-5.848 to 3.102	0.548	-2.236	-7.503 to 3.031	0.405
Not work	Reference			Reference			Reference			Reference		
Monthly income												
0 to 4k	-4.560	-10.281 to 1.161	0.118	.380	-1.144 to 1.904	0.625	.072	-1.393 to 1.537	0.923	-.210	-1.934 to 1.515	0.812
4,001 to 8k	.439	-4.127 to 5.004	0.851	.352	-.864 to 1.568	0.571	.422	-.747 to 1.591	0.479	.438	-.938 to 1.813	0.533
8,001 to 12k	-1.109	-6.059 to 3.841	0.661	.266	-1.053 to 1.584	0.693	.493	-.774 to 1.761	0.446	.386	-1.106 to 1.878	0.612
12,001 to 16k	-2.261	-7.286 to 6.202	0.418	-765	-.692 to 2.221	0.304	.244	-1.156 to 1.643	0.733	.442	-1.206 to 2.090	0.599
>16k	-2.356	-8.235 to 4.284	0.255	-.047	-1.844 to 1.749	0.959	.332	-1.395 to 2.059	0.707	-.265	-2.298 to 1.768	0.798
Not work	Reference			Reference			Reference			Reference		
Risk of loss job												
Yes	5.588	-9.853 to 21.030	0.478	2.219	-1.985 to 6.242	0.310	-.912	-4.855 to 3.030	0.650	1.691	-2.963 to 6.345	0.476
No	-3.107	-18.360 to 12.145	0.690	.288	-3.775 to 4.352	0.889	-.993	-4.896 to 2.910	0.618	-.218	-4.815 to 4.379	0.926
Not work	Reference			Reference			Reference			Reference		
Reduction in working days												
Yes	2.817	-12.580 to 18.215	0.720	.322	-3.780 to 4.423	0.878	2.973	-.981 to 6.927	0.141	.707	-3.933 to 5.348	0.765
No	.678	-14.566 to 15.921	0.931	-.469	-4.530 to 3.591	0.821	1.227	-2.678 to 5.133	0.538	.376	-4.218 to 4.970	0.873
Not work	Reference			Reference			Reference			Reference		
Monthly income reduction												
Yes	-2.709	-7.153 to 1.735	0.232	-.452	-1.636 to .732	0.454	-1.069	-2.207 to .068	0.065	-1.008	-2.348 to .331	0.140
No	-3.280	-7.453 to .892	0.123	-.499	-1.495 to .728	0.499	-.893	-1.961 to .176	0.101	-1.061	-2.319 to .197	0.098
Not work	Reference			Reference			Reference			Reference		

Abbreviation: β, beta coefficient; CI, confidence interval

### Physical Health Status, Psychological Distress, and Mental Health

The results evidenced that 37.4% respondents rated their health as excellent, 43.01% as very good, 15.1% as good, 4.47% as regular, and 0.01% as bad. Around 2.8% of respondents had consulted a doctor in the clinic; 2.8% had been admitted to the hospital; 2% had been tested for COVID-19; and 4.4% had been under quarantine by a health authority. However, clinic consultations, hospitalizations, being placed in obligatory quarantine, health status, and being tested for COVID-19 were not associated with DASS-21 and IES-R scores. Additionally, 54% participants reported the presence of physical symptoms of COVID-19 during the past 14 days, most frequently headache (48.71%), cough (14.01%), coryza (10.76%), and fever (8.37%). Finally, the statistical analysis showed a significant association between the presence of physical symptoms with higher scores in the scales of the IES-R (B = 6.609; 95% CI = 4.355 to 8.863) and the subscales of the DASS-21 (depression: B = 1.805; 95% CI = 1.198 to 2.412; anxiety: B = 2.658; 95% CI = 2.090 to 3.227; and stress B = 2.688, 95% CI = 2.025 to 3.352) (see Table 4).

**TABLE 4:** Association Between Physical Health Status in the Past 14 Days and the Psychological Impact and Mental Health Status During the Epidemic

	Impact of Event Scale Revised (IES-R)			Depression, Anxiety and Stress Scales (DASS-21)								
	IES-R Total			Depression			Anxiety			Stress		
	*β*	95% CI	*P* value	*β*	95% CI	*P* value	*β*	95% CI	*P* value	*β*	95% CI	*P* value
Self-rated physical health status												
Bad	13.529	-23.096 to 50.153	0.469	-3.316	-12.903 to 6.271	0.498	2.474	-6.563 to 11.511	0.524	-1.978	-12.641 to 8.685	0.716
Regular	18.242	11.140 to 25.344	0.076	7.835	5.976 to 9.694	0.061	7.464	5.712 to 9.217	0.692	7.126	5.059 to 9.194	0.125
Good	8.936	5.474 to 12.398	0.098	3.710	2.804 to 4.616	0.223	2.940	2.085 to 3.794	0.235	3.224	2.217 to 4.232	0.098
Very good	1.740	-.800 to 4.280	0.179	1.181	.517 to 1.846	0.145	1.199	.572 to 1.826	0.114	1.410	.670 to 2.149	0.823
Excellent	Reference			Reference			Reference			Reference		
Clinical consultation												
Yes	-.411	-19.021 to 18.200	0.965	-3.819	-8.691 to 1.052	0.124	-1.275	-5.867 to 3.317	0.586	-2.151	-7.570 to 3.267	0.436
No	Reference			Reference			Reference			Reference		
Hospitalization												
Yes	4.557	-14.047 to 23.161	0.631	3.536	-1.334 to 8.406	0.155	1.694	-2.896 to 6.285	0.469	2.461	-2.956 to 7.877	0.373
No	Reference			Reference			Reference			Reference		
Being quarantined by a health authority												
Yes	2.624	-2.748 to 7.988	0.3388	.106	-1.301 to 1.512	0.883	.628	-.698 to 1.954	0.353	.331	-1.233 to 1.895	0.678
No	Reference			Reference			Reference			Reference		
Being tested for COVID-19												
Yes	1.325	-1.138 to -.367	0.245	.736	-.536 to -.294	0.294	.134	-.495 to -.109	0.837	.513	-.1.796 to -.898	0.492
No	Reference			Reference			Reference			Reference		
Physical symptoms												
Yes	6.609	4.355 to 8.863	0.0001*	1.805	1.198 to 2.412	0.0001**	2.658	2.090 to 3.227	0.0001**	2.688	2.025 to 3.352	0.0001**
No	Reference			Reference			Reference			Reference		

Abbreviations: β, beta coefficient; CI, confidence interval.

*P<0.05

**P<0.001

### Contact History, Psychological Distress, and Mental Health

Overall, 2.7% of respondents had been in direct contact with an individual with confirmed COVID-19; 3.4% showed indirect contact with an individual with confirmed COVID-19; and 8.1% reported indirect contact with an individual with confirmed COVID-19. Respondents with a history of direct contact with a confirmed case of COVID-19 showed a significant association with depression (B = 2.168; 95% CI = 3.677 to 4.330), anxiety (B = 2.905; 95% CI = 1.111 to 4.699), and stress (B = 2.499; 95% CI = 0.414 to 4.584). Finally, persons who had indirect contact with an individual with confirmed COVID-19 evidenced a significant association with anxiety (B = 2.198; 95% CI = 0.582 to 7.103). The contact history of COVID-19 was not associated with IES-R scores (see Table 5).

**TABLE 5:** Association Between Contact History in the Past 14 Days and the Mental Health Status and Psychological Impact of the COVID-19 Outbreak

	Impact of Event Scale Revised (IES-R)			Depression, Anxiety and Stress Scales (DASS-21)								
	IES-R Total			Depression			Anxiety			Stress		
	*β*	95% CI	*P* value	*β*	95% CI	*P* value	*β*	95% CI	*P* value	*β*	95% CI	*P* value
Direct contact with an individual with confirmed COVID-19												
Yes	5.357	-1.641 to 12.355	0.133	2.168	-.286 to 4.051	0.024*	2.905	1.111 to 4.699	0.002*	2.499	.414 to 4.584	0.0019*
Not	Reference			Reference		.	Reference			Reference		.
Indirect contact with an individual with confirmed COVID-19												
Yes	5.343	-.963 to 4.853	0.097	1.680	-.017 to 3.377	0.062	2.198	.582 to 3.815	0.008*	1.185	-.693 to 3.064	0.216
No	Reference			Reference			Reference			Reference		
Direct contact with an individual with suspected COVID-19												
Yes	.806	-3.396 to 2.434	0.707	0.076	-1.055 to 1.206	0.895	-.071	-1.148 to 1.007	0.898	.267	-.984 to 1.519	0.679
No	Reference			Reference			Reference			Reference		

Abbreviations: β, beta coefficient; CI, confidence interval.

### Knowledge and Concerns, Psychological Distress, and Mental Health

A large number of the respondents knew the means of transmission of COVID-19 (94.5%); however, 49.5% lacked confidence related to the security of COVID-19 test. The majority of respondents (51.7%) were very satisfied or satisfied with the amount of health information available on COVID-19, 35.5% participants were just satisfied, and 13.8% were not satisfied. In addition, 35.4% of respondents were very satisfied or satisfied with the amount of information that the government provided regarding new cases and treatments tendency about COVID-19, and 64.6% considered their level of satisfied was regular or no satisfied. The main sources of information about COVID-19 were social networks (Facebook/Twitter, 34.8%), television (27.9%), and scientific journals (15.2%).

Knowledge about transmission was significantly associated with higher scores in DASS-21 subscales (depression: B = -2.058; 95% CI = -3.373 to -.743; anxiety: B = -2.546; 95% CI = -3.811 to 1.281; and stress: B = -1.847; 95% CI = -3.307 to -0.388) and IES-R scale (B = -5.439; 95% CI = -10.342 to -.536). Similarly, lack confidence related to security on COVID-19 test was significantly associated with higher scores in DASS-21 subscales (depression: B = -1.027; 95% CI = -1.658 to -0.396; and stress: B = -1.029; 95% CI = -1.730 to -0.329) and IES-R scale (B = -2.588; 95% CI = -4.930 to -0.247).

Satisfaction level about the amount of health information available about COVID-19 was significantly associated with higher scores in DASS-21 depression subscale. No associations were found between the satisfaction about the amount health information and IES-R scores. Finally, the level of satisfaction about amount information that the government provided regarding new cases and treatments of COVID-19 was significantly associated both higher scores in DASS-21 (anxiety and stress subscale) and IES-R scale. Social network, as the main source of information about COVID-19, was significantly associated with a higher IES-R scale score (B = 3.419; 95% CI = 0.613 to 6.226) (see Table 6).

**TABLE 6:** Association Between Knowledge and Concerns About the COVID-19 Outbreak and the Psychological Impact and Mental Health Status

	Impact of Event Scale Revised (IES-R)			Depression, Anxiety and Stress Scales (DASS-21)								
	IES-R Total			Depression			Anxiety			Stress		
	*β*	95% CI	*P* value	*β*	95% CI	*P* value	*β*	95% CI	*P* value	*β*	95% CI	*P* value
Knowledge about ways of transmission												
Yes	-5.439	-10.342 to -.536	0.030*	-2.058	-3.373 to -.743	0.002*	-2.546	-3.811 to 1.281	0.0001**	-1.847	-3.307 to -.388	0.013*
Not	Reference			Reference		.	Reference		.	Reference		.
Confidence in diagnosis COVID-19												
Yes	-2.588	-4.930 to -.247	0.030*	-1.027	-1.658 to -.396	0.0001**	-.496	-1.104 to .11	0.109	-1.029	-1.730 to -.329	0.004*
No												
Level of satisfaction of health information												
No satisfaction	2.216	-4.939 to 9.371	0.544	2.766	.830 to 4.702	0.0001**	1.679	-.182 to 3.541	0.077	1.294	-.854 to 3.442	0.238
Poor satisfaction	-1.652	-7.120 to 3.815	0.554	2.153	.676 to 3.630	0.005*	1.385	-.036 to 2.805	0.056	1.549	-.099 to 3.178	0.066
Neutral	.366	-3.717 to 4.449	0.861	1.241	.138 to 2.345	0.027*	1.001	-.060 to 2.062	0.065	1.063	-.162 to 2.287	0.089
Satisfied	-.764	-4.762 to 3.125	0.700	.895	-.154 to 1.944	0.094	.352	-.657 to 1.360	0.495	.752	-.412 to 1.915	0.206
Very satisfied	Reference			Reference			Reference			Reference		
Level of satisfaction of information about the trend of new cases and potential treatment for COVID-19												
No satisfaction	11.120	5.212 to 17.028	0.0001**	1.092	-.505 to 2.689	0.180	1.628	.092 to 3.163	0.038*	2.881	1.109 to 4.653	0.0001**
Poor satisfaction	7.687	2.442 to 12.931	0.004*	.988	-.424 to 2.400	0.170	1.617	.259 to 2.975	0.020*	1.978	.411 to 3.545	0.013*
Neutral	8.068	3.521 to 12.615	0.0001**	1.047	-.182 to 2.276	0.095	1.092	-.090 to 2.274	0.070	1.731	.367 to 3.095	0.013*
Satisfied	4.826	.256 to 9.396	0.038*	.357	-.879 to 1.593	0.572	.760	-.429 to 1.949	0.210	1.158	-.214 to 2.529	0.098
Very satisfied	Reference			Reference			Reference			Reference		
Main source of information												
Social networks	3.419	.613 to 6.225	0.017*	1.092	-.505 to 2.689	0.180	1.628	.092 to 3.163	0.038	2.881	1.109 to 4.653	0.142
Parents and friends	3.744	-3.502 to 10.989	0.311	.988	-.424 to 2.400	0.170	1.617	.259 to 2.975	0.087	1.978	.411 to 3.545	0.253
Internet	28.249	-8.189 to 64.688	0.129	1.047	-.182 to 2.276	0.095	1.092	-.090 to 2.274	0.070	1.731	.367 to 3.095	0.472
Newspaper and magazines	.932	-3.211 to 5.076	0.659	.357	-.879 to 1.593	0.572	.760	-.429 to 1.949	0.210	1.158	-.214 to 2.529	0.098
Scientific journals	-6.542	-15.393 to 2.309	0.147	-2.525	-.654 to 1.093	0.763	.374	-.237 to 1.984	0.482	1.035	-.353 to 1.948	0.948
Television	Reference			Reference			Reference			Reference		

Abbreviations: β, beta coefficient; CI, confidence interval.

*P<0.05

**P<0.001

### Precautionary Measures, Psychological Distress, and Mental Health

The main precautionary measures about COVID-19 were hand washing behavior (35.74%), home confinement (28.77%), and avoid handshake (16.10%). Statistical analysis showed that washing hands was significantly associated with higher scores in the IES-R (B = 0.49; 95% CI = 0.86 to 0.015), and the DASS stress (B = 0.125; 95% CI = 0.52 to 0.09), anxiety (B = 0.82; 95% CI = 0.65 to 0.21), and depression subscales (B = 0.45; 95% CI = 0.8 to 0.17). Similarly, wearing masks was significantly associated with higher lower IES-R scores (B = 0.63; 95% CI = 0.07 to 1.12) and lower scores in the DASS anxiety (B = 0.54; 95% CI = 0.74 to 0.08) and depression subscales (B = 0.41; 95% CI = 0.71 to 0.09). The majority of participants spent >17 h/d at home (66.2%). Regarding assessment with the IES-R, respondents that stay at home >9 h showed a significant association with higher scores in the scale IES-R and the DASS-2 depression subscale (see Table 7).

**TABLE 7:** Association Between Precautionary Measures in the Past 14 Days and the Psychological Impact and Mental Health Status

	Impact of Event Scale Revised (IES-R)			Depression, Anxiety and Stress Scales (DASS-21)								
	IES-R Total			Depression			Anxiety			Stress		
	*β*	95% CI	*P* value	*β*	95% CI	*P* value	*β*	95% CI	*P* value	*β*	95% CI	*P* value
Precautionary measures												
Cover mouth	7.660	-11.709 to 27.028	0.438	-.149	.265 to .178	0.738	-2.384	-2.589 to -1.389	0.843	-5.256	-3.498 to -2.589	0.783
Wash hands	.490	.860 to .015	0.0001**	.450	.800 to .170	0.001*	.820	.650 to .210	0.030*	.125	.520 to .009	0.040*
Wear mask	.630	.007 to 1.120	0.020*	.410	.710 to .009	0.003*	.054	.740 to .080	0.0001**	-2.489	-2.193 to -1.848	0.582
Avoid buying food	23.227	-5.859 to 52.313	0.118	-1.482	.849 to .184	0.277	-1.134	-1.284 to .879	0.247	-1.084	-.875 to -.583	0.257
Avoid handshakes	-18.796	-40.044 to 2.451	0.083	-.893	.746 to .198	0.175	-.897	-.746 to -.264	0.097	-.248	-.763 to - .384	0.176
Avoid leaving home	2.295	-15.222 to 19.812	0.797	-9.092	-27379 to 9.196	0.330	-.521	-.347 to .183	0.273	-.198	-1.837 to -.266	0.174
None	Reference			Reference			Reference			Reference		
Hours staying at home per day												
0 to 8	-1.583	-2.913 to 1.485	0.183	-3.582	-2.847 to -.183	0.098	-2.487	-2.103 to -1.829	0.914	-.382	-.234 to -.129	0.094
9 to 16	-3.381	-2.583 to -.660	0.015*	-5.389	-1.385 to -.857	0.040*	-1.837	-1.847 to -.1102	0.728	-.155	-.748 to - .384	-0.233
>17	-2.165	-2.198 to -.245	0.004*	-4.258	-1.982 to -.218	0.002*	-1.248	-1.502 to -.837	0.148	-.140	-.893 to -.129	-0.104

## DISCUSSION

This study examined the psychological distress of COVID-19 in a Mexican sample, just 1 wk after a national health emergency was declared in Mexico. To our knowledge, this study is the first to evaluate psychological distress in a Mexican sample. Our findings showed that 50.3% of respondents rated the psychological distress of the outbreak as moderate to severe; 15.7% of respondents reported moderate to severe depressive symptoms; 22.6% of respondents reported moderate to severe anxiety symptoms; and 19.8% reported moderate to severe stress levels. The prevalence of moderate or severe psychological distress as measured by IES-R was higher than the prevalence of depression, anxiety, and stress as measured by the DASS-21. The differences in the results obtained between IES-R and DASS-21 may be due to the fact that the IES-R assesses psychological distress after a specific event. Therefore, respondents might refer the COVID-19 outbreak as the event, while the DASS-21 did not specify any such event, as previously reported.^20^

According to similar epidemics and pandemics, in such cases, serious concerns, such as fear of death can arise, and feelings of loneliness and anger can develop among people who are quarantined.^26^ In addition, people who are quarantined lose face-to-face connections and traditional social interventions, and this is a stressful phenomenon.^27^ Therefore, at these points, it was not surprising to find that the Mexican sample showed severe psychological distress during the COVID-19 outbreak, similarly with the findings originating from China,^20,28^ Canada,^29^ Iran,^27^ Japan,^30^ and Singapore.^31^

In support, it is recognized that widespread outbreaks of infectious diseases, such as SARS,^32^ H1N1,^33^ and actually, COVID-19, are associated with psychological distress and symptoms of mental illness.^34^ Both psychologists, psychiatrists, and the health care system at large should be aware of these manifestations to subsequently implement early psychological interventions according the needs of specific populations.

Our findings suggest that females suffered greater psychological distress of the outbreak as well as higher levels of stress, anxiety, and depression. This finding corresponds to previous studies which found that women were at higher risk of depression,^35,36^ anxiety,^37^ and stress.^38^ It is likely that genetic, biological, chemical, hormonal, environmental, psychological, and social factors all intersect to contribute to mental disorders in women.^39^ In addition, we found that with older age, participants showed greater stress and had a greater psychological distress of COVID-19 outbreak. Young, middle-aged, and older adults may have diﬀerent levels of exposure to school-related, work-related, and health-related stressors because of their life stages and associated life events.^40^ In addition, the Mexico government has shut down schools at all levels indefinitely. Currently, teachers are using online platforms to deliver lectures or other teaching activities. However, the uncertainty and potential negative distress on academic progression could have an adverse effect on the mental health of young people.

We found that divorced status had a greater likelihood of depression during the epidemic; in support, prior findings showing that the end of marriage through divorce is associated with a significant increase in the probability of a future depressive episode.^41^ Participants who live with 1 other person exhibited greater anxiety and greater psychological distress of the COVID-19 outbreak. This finding corresponds to previously extensive epidemiological studies which found that family size predicts anxiety.^42^

On the other hand, it is necessary that the health authorities identify the immediate psychological needs of the general population presenting with physical symptoms during the epidemic. Our results revealed that the Mexican sample, which presented specific symptoms of COVID-19, experienced greater psychological distress on the outbreak and higher levels of stress, anxiety, and depression. Health professionals should take the opportunity to provide resources for psychological support and interventions for those who present with the above symptoms, especially during clinical consultations. Moreover, such interventions should also be guaranteed for infected individuals and their caregivers.^20^ Taking a family history is essential, and health professionals should enquire about the level of concern for other family members of contracting COVID-19, as these concerns are associated with altered mental status. In support, our results showed respondents had been in direct or indirect contact exhibited higher levels of anxiety, stress, and depression.

The goal of communication before and during a pandemic is to provide and exchange relevant information with the public, partners, and stakeholders to allow them to make well-informed decisions and take appropriate actions to protect health and reduce the distress of rumors.^43^ In our study, lack of confidence in the COVID-19 test security and lower satisfaction with the health information received were associated with higher psychological distress of the outbreak and higher levels of stress, anxiety, and depression. Therefore, it is crucial to provide clear health information, with comprehensible messages and based on evidence to avoid adverse psychological reactions.^44^

A major adverse consequence of the COVID-19 pandemic is likely to be increased social isolation and loneliness, which are strongly associated with anxiety, depression, self-harm, and suicide attempts across the lifespan.^45^ In our study, the majority of the participants that were homebound for >9 h/d during the epidemic suffered greater psychological distress from the outbreak as well as higher levels of depression. Under these conditions, the content of psychological interventions needs to be modified to suit the needs of the general population during the epidemic. Psychologists should produce and disseminate information materials on the psychological consequences of the quarantine and toll-free numbers should be activated to support all those who are confronted with psychological distress.^46^ For example, cognitive behavior therapy could be delivered online or by means of telephone to counteract depression in the home environment. For this reason, fine tuning effective psychological interventions is a future need, given that, after the public health emergency, a psychological distress emergency seems on the horizon.

Finally, our findings showed that specific precautionary measures as hand hygiene and wearing masks were associated with lower levels of psychological distress, depression, anxiety, and stress. Our finding is similar to a previous study, which suggests that the precautionary measures adopted to prevent the spread of COVID-19 could have had protective psychological effects during the early stage of the epidemic.^20^

This study has several limitations. First, due the time-sensitivity of the COVID-19 outbreak, we used the snowball sampling strategy; this strategy was not based on a random selection of the sample, and the study population did not reflect the actual pattern of the general population. Second, the sampling was carried out by students, leading to selection bias. Therefore, our conclusion was less generalizable to the entire population, particularly less-educated people. Last, the number of respondents with contact history and who had sought medical consultations was very small. As a result, our findings could not be generalized to confirmed or suspected cases of COVID-19.

Notwithstanding the above limitations, this study provides invaluable information on the psychological effects in the Mexican sample, 1 wk after the outbreak of COVID-19 was declared a national health emergency in Mexico. In addition, our results could be used as a historical reference and provide a baseline for evaluating prevention, control, and treatment efforts throughout the remainder of the COVID-19 epidemic.

## CONCLUSIONS

Together, the data show that the COVID-19 outbreak results in considerable psychological distress among the Mexican sample examined. Public health officials, infectious diseases physicians, psychiatrists, psychologists, and the health care system at large need to be made aware of this issue.

## Supplementary material

For supplementary material accompanying this paper visit http://dx.doi.org/10.1017/dmp.2020.215. click here to view supplementary material
