# Supplementary material for: Psychological Effects and Associated Factors of COVID-19 in a Mexican Sample
Source: Disaster Med Public Health Prep. 2020 Jun 24:1–12. doi: 10.1017/dmp.2020.215 (PMC7385317; doi:10.1017/dmp.2020.215)

**Appendix 1: Questionnaire and answers**

| **AREA:** Demographic data | |
| --- | --- |
| **QUESTIONS** | **ANSWERS** |
| Sex | - Female - Male |
| Age | - 18-28 - 29-39 - 40-50 - 51-61 - +62 |
| Level of education | - Primary school - Secondary school - High school - College - Specialty - Master - Doctorate |
| Residential location | - State |
| Marital status | - Single - Common-law marriage - Married - Divorced - Widowed |
| Parental status | - With children - Not children |
| Household size | - 1 - 2 - 3 - >3 |

| **AREA:** Financial status | |
| --- | --- |
| **QUESTIONS** | **ANSWERS** |
| Employment status | - Student - Housewife - Employee - Unemployed - Retired |
| Monthly income | - 0 to 4k - 4,001 to 8k - 8,001 to 12k - 12,001 to 16k - >16k - Not work |
| Risk of loss job | - Yes - No - Not work |
| Reduction in working days | - Yes - No - Not work |
| Monthly income reduction | - Yes - No - Not work |

| **AREA:** Physical health | |
| --- | --- |
| **QUESTIONS** | **ANSWERS** |
| Presence of symptoms such as: fever, chills, headache, myalgia, cough, difficulty in breathing, dizziness, coryza and sore throat | - Yes - No |
| Self-rating physical health status | - Bad - Regular - Good - Very good - Excellent |
| Consultation with a doctor  *Means* the respondent gone to doctor public or private for any symptoms of Covid-19. | - Yes - No |
| Admission to the hospital  *Means*: means the respondent was hospitalized in public or private medical related to COVID-19. | - Yes - No |
| Being quarantined by a health authority | - Yes - No |
| Being tested for COVID-19 | - Yes - No |
| Presence of symptoms such as: fever, chills, headache, myalgia, cough, difficulty in breathing, dizziness, coryza and sore throat | - Yes - No |

| **AREA:** Contact history | |
| --- | --- |
| **QUESTIONS** | **ANSWERS** |
| Direct contact with an individual with confirmed COVID-19 | - Yes - No |
| Indirect contact with an individual with confirmed COVID-19 | - Yes - No |
| Direct contact with an individual with suspected COVID-19 | - Yes - No |

| **AREA:** Knowledge and concerns | |
| --- | --- |
| **QUESTIONS** | **ANSWERS** |
| Knowledge about the routes of transmission | - Yes - No |
| Confidence in diagnosis | - Yes - No |
| Level of satisfaction of health information about COVID-19 | - No satisficed - Poor satisficed - Regular - Satisficed - Very satisficed |
| Level of satisfaction of information about the trend of new cases and the potential treatment for COVID-19 infection | - No satisficed - Poor satisficed - Regular - Satisficed - Very satisficed |
| Mainly source of information | - Social networks - Parents and friends - Internet - Newspaper and magazines - Scientific journals - Television |
| Knowledge about the routes of transmission | - Yes - No |
| Confidence in diagnosis | - Yes - No |

| **AREA:** Precautionary measures | |
| --- | --- |
| **QUESTIONS** | **ANSWERS** |
| Covering mouth when coughing and sneezing | - Yes - No |
| Washing hands with soap | - Yes - No |
| Washing hands immediately after coughing or sneezing | - Yes - No |
| Wearing a mask regardless of the presence or absence of symptoms | - Yes - No |
| Avoid buying food in informal establishments | - Yes - No |
| Avoid handshakes and leaving home only in indispensable situations | - Yes - No |
| Covering mouth when coughing and sneezing | - Yes - No |

**Appendix 2: Frequency distribution of the respondents of Mexican sample**


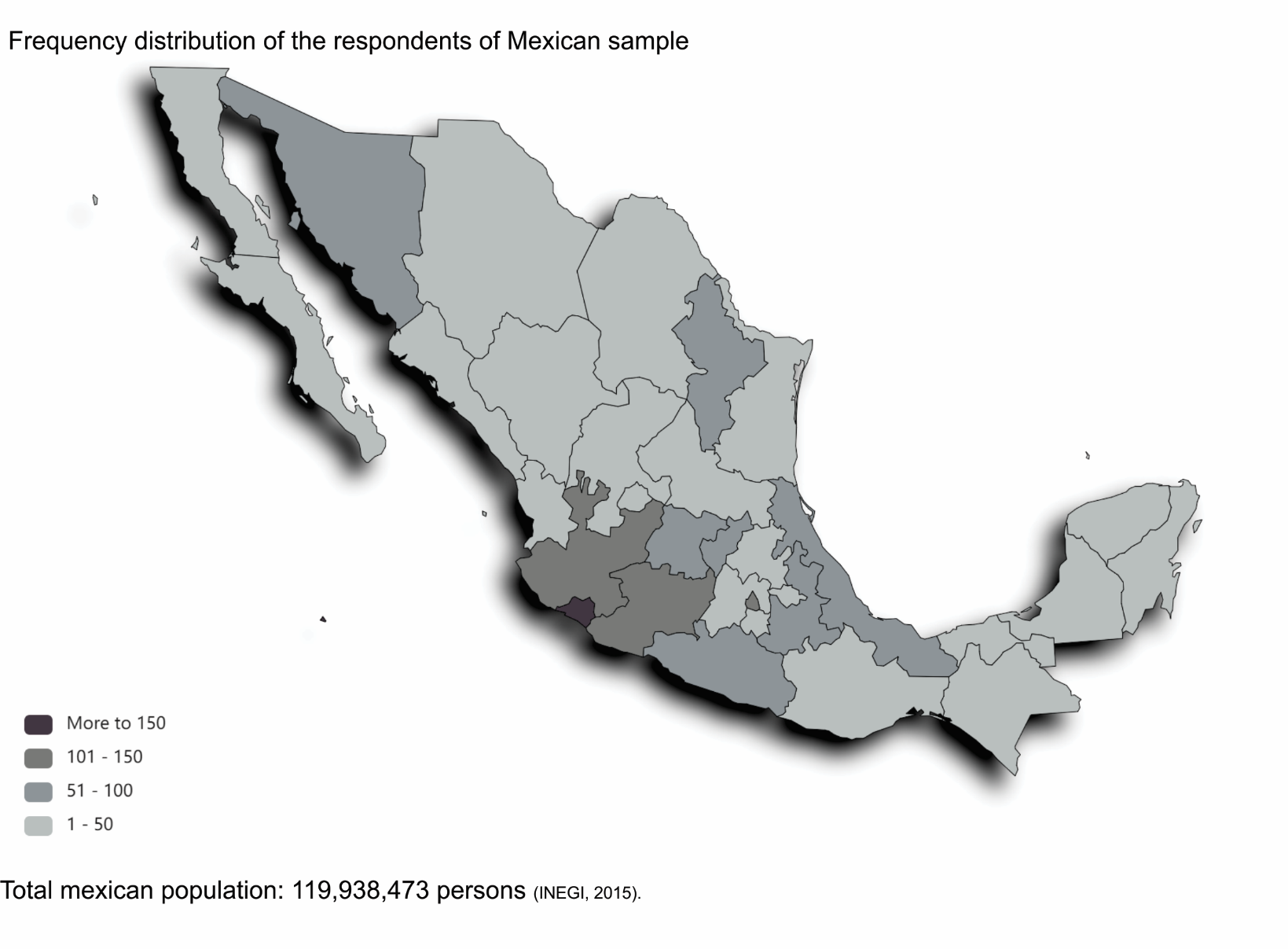

Supplement: Supplementary file 1 [file S1935789320002153sup001.docx]
